# Humour Against Binge Drinking During the COVID-19 Pandemic: A Cartoon-Based Anti-Alcohol Health Campaign Targeting Women-Who-Have-Sex-With-Women

**DOI:** 10.1007/s41042-022-00068-0

**Published:** 2022-07-14

**Authors:** Hedy Greijdanus, Maaike van der Voorn

**Affiliations:** Heymans Institute for Psychological Research, University of Groningen, Grote Kruisstraat 2/1, 9712 TS Groningen, The Netherlands

**Keywords:** Humor, Health, Inclusion, Alcohol abuse, Emotions, LGBTQ, COVID-19, Social identity

## Abstract

This study focuses on the role of humour in health and well-being of women-who-have-sex-with-women (WSW) during COVID-19. This group has been shown to be vulnerable to alcohol abuse, especially as self-medication coping with social consequences of the COVID-19 pandemic. We investigated the potential usefulness of WSW-inclusive (i.e., depicting a female-female romantic couple) versus hetero-normative (i.e., depicting a male-female romantic couple) humorous cartoons in an anti-alcohol health campaign against excessive drinking among WSW. One-hundred-and-twenty-seven self-categorized WSW of diverse genders (woman, non-binary) and sexual orientations (e.g., lesbian, bisexual, pansexual, queer, heterosexual) participated in a 2 × 2 factorial between-participants design. Data were collected during the first months of the COVID-19 pandemic in The Netherlands. Humorous cartoons explicitly referring to lockdown measures systematically varied the humour subject (punchline about excessive drinking versus staying sober) and the couple (male-female, female-female) that were depicted. Although the (very brief) health message did not influence binge drinking determinants, the humorous health campaign depicting a female-female couple was perceived as more inclusive and evoked more amusement and less anger than when the cartoons depicted a male-female couple. High WSW identifiers were less amused about the health campaign text (but not the cartoons), less likely to share campaign materials offline (but not online), and had more positive binge drinking attitudes but lower binge drinking intentions than low identifiers. Implications are discussed.

In December 2019, the first human infections were discovered with the new coronavirus SARS-CoV-2, which causes acute respiratory syndrome COVID-19 in humans (Zhou et al., 2020). This disease spread at an unprecedented rate across the rest of the world, and this pandemic placed a heavy burden on health systems worldwide (Haldane et al., 2021; Verity et al., 2020). In anticipation of medical interventions, governments around the world have implemented various behavioural interventions to limit contact between people in order to slow down the spread of the virus (Perra, 2021). One way in which people cope with the COVID-19 pandemic and its consequences is via humour, by sharing memes through social media (Strick, 2021). Alternatively, they may develop more destructive means such as binge drinking to soften the edges of solitary confinement and social isolation. Recent research indicates that sexual minority women may be particularly vulnerable to such destructive coping (Cerezo et al., 2021) and that there is a need for health messages specifically targeting vulnerable, marginalized LGBTQ + groups (Drabble & Eliason, 2021). In the current research, we combine these timely topics by investigating whether humour around the COVID-19 pandemic can play a role in increasing perceived inclusion and preventing binge drinking (i.e., consuming > 4 drinks on a single occasion) among women who have sex with women (WSW). Can a humorous health campaign thus make people more resilient and help them to function more adaptively in the face of the demands that life can pose?

We start by briefly outlining some ways in which the COVID-19 pandemic and its countermeasures may have detrimental consequences for health and wellbeing, particularly among WSW. Then we will turn to our core argument by describing how, in the current view, a humorous health campaign can constructively contribute to WSW’s resilience. Building on existing theorizing and empirical findings regarding social identity and health behaviours, we propose that such humour use may be beneficial in three ways. First, it may improve perceived inclusion among the marginalized group of WSW. Second, we propose that it can lead to more positive reception of the health campaign and wider dissemination of the campaign message. And third, it may ultimately reduce binge drinking in this community. Moreover, we examine whether social identification with WSW strengthens the proposed beneficial effects of humour use on these outcomes.

## Detrimental health and wellbeing consequences of (measures against) COVID-19

In response to the COVID-19 pandemic, countries around the world took measures to mitigate the spread of the virus. Many governments introduced social distancing measures or lockdowns, to limit citizens’ contact outside their own households. These measures were intended to benefit citizens’ health but research also have potential negative side effects. Indeed, quarantine can induce a range of negative stress-related psychological consequences (for a review, see Brooks et al., 2020). More specifically, measures that limit social connectedness can result in loneliness – and loneliness in turn, through increased stress, affects alcohol use (Segrin et al., 2018). Moreover, loneliness can exacerbate existing mental health difficulties or increase the likelihood of depression (Hoffart et al., 2020; Lasgaard et al., 2011). Self-medication with alcohol is one of the ways in which people may cope with these mental health issues (Turner et al., 2018). Thus, not only stress from the pandemic itself but also the measures to contain and limit further spreading of the virus may have indirectly led people to reach for the bottle.

Corroborating this rationale, US adults showed increases in both mental health problems and alcohol use since the COVID-19 pandemic (Horigian et al., 2020) and time spent at home during the pandemic was associated with their increase in binge (Weerakoon et al., 2020). Australian data links adults’ increased alcohol intake since COVID-19 to more severe depression or anxiety symptoms (Tran et al., 2020). Zooming in on consequences of social distancing measures, a survey among Canadian adolescents showed an increase in their alcohol consumption after these measures were introduced (Dumas et al., 2020). Alcohol use likewise increased due to the COVID-19 pandemic among Russian and Belarusian students, especially among those in self-quarantine (Gritsenko et al., 2020). A large-scale longitudinal study revealed that UK citizens’ stress levels and alcohol use increased during lockdown, especially among women and young adults (Niedzwiedz et al., 2020). A Polish longitudinal study paints a more nuanced picture, indicating that only relatively heavy drinkers increased their alcohol consumption because of the pandemic (Chodkiewicz et al., 2020). All in all, there seems to be a general concern that lockdown and social distancing measures against COVID-19 may invigorate alcohol abuse (for a brief review, see Ramalho 2020).

The current study specifically targets binge drinking among women who have sex with women (WSW). This approach squares with existing evidence of sexual minority women’s vulnerability to excessive drinking (Cerezo et al., 2020). This vulnerability is further corroborated by a large-scale self-report study among Australian WSW (Hyde et al., 2009). WSW drink more and more often than women in the general population. Moreover, although a quarter reported to binge drink at least once a week, only a small minority saw themselves as heavy drinkers. These numbers highlight not only that heavy drinking is a problem among WSW but also that this problematic behaviour is perceived as normal. Thus, it seems that traditional campaigns have been found wanting in fighting alcohol problems among WSW, as illustrated by the high vulnerability and prevalence of heavy drinking in this group. One reason for the lack of effectiveness may have been that health campaigns typically target the general population and that specific tailoring for sexual minorities is mostly done in health campaigns on issues such as safe sex. It is vital that health campaigns on binge drinking explicitly target high-risk groups (Shields et al., 2016) and in particular vulnerable LGBTQ + groups (Drabble & Eliason, 2021).

## Social identity, perceived inclusion, and collective resilience among WSW

LGBTQ + minorities such as WSW have been shown to use alcohol as a means of coping particularly with the COVID-19 pandemic (Cerezo et al., 2021) as well as coping with sexual orientation discrimination more generally (Cerezo et al., 2020; McCabe et al., 2010). Indeed, decades of research from the social identity approach show accumulating evidence that people are motivated to maintain a positive feeling about the groups they belong to (for an overview, see Postmes & Branscombe 2010). People who strongly identify with a social group are more committed to it (Ellemers et al., 1997) and are more likely to pursue social change to improve their group’s collective conditions (Van Zomeren et al., 2008). Discrimination of social groups can thwart group members’ need for a positive social identity and has detrimental consequences for wellbeing (Schmitt et al., 2014). Moreover, minorities such as LGBTQ + are frequently merely tolerated (i.e., not openly discriminated) rather than truly accepted and included, and this mere tolerance can also decrease wellbeing (Bagci et al., 2020). This implies that a campaign aimed at improving WSW’s resilience should explicitly make them feel included. Research on diversity campaigns in organizations shows that job applicants belonging to a cultural majority feel more included if the company’s diversity message explicitly mentions the cultural majority group (Jansen et al., 2015). Thus, explicitly mentioning a certain social identity in health campaign messages could increase perceived inclusion among members of that social group.

Social identities play an important role in people’s health-related norms and behaviours, so destructive or constructive group norms can encourage, respectively, destructive or constructive health behaviours (for an overview, see Haslam et al., 2009). So, on the one hand binge drinking behaviours may be seen as normal among WSW (Cerezo et al., 2020; Hyde et al., 2009). This may seem to suggest that the social identity of WSW is at the core of the problem. On the other hand, however, a growing body of evidence on collective resilience during the COVID-19 pandemic suggests that social identities and communities should be considered as the solution rather than the problem in times of crisis (Drury et al., 2021; Reicher & Bauld, 2021). Collective resilience refers to how collectives of people, rather than individuals, respond to the challenges they face with solidarity and cohesion (Drury et al., 2009; Williams & Drury, 2009). Leaders can invoke collective resilience among their followers to face the challenges of the COVID-19 pandemic by harnessing social identities (Vignoles et al., 2021). In a similar vein, a health campaign may speak to a specific social identity to fuel collective resilience and stimulate adaptive and supportive feelings, perceptions, and behaviours. Indeed, health promotion for LGBTQ + people requires active acknowledgement and affirmation of their identities (Valdiserri et al., 2019).

## Humour as a valuable component in health campaigns

Knowledge on maladaptive alcohol consumption during the COVID-19 pandemic evidently is still emergent at the moment of writing. However, more is known about prevention of alcohol abuse and about the effectiveness of health campaigns generally. Two prominent theoretical frameworks that have been used to explain health behaviours are the health belief model (HBM) and the theory of planned behaviour (TPB). According to the HBM (Rosenstock, 1974), people decide whether or not to engage in health behaviours based on their perceptions of the probability of a negative health outcome, the severity of negative consequences, the effectiveness of the health behaviour to prevent negative outcomes, obstacles to engage in the health behaviour, and cues to action. This latter concept refers to reminders to engage in the health behaviour, for instance persuasive appeals by close others or health campaigns. The TPB (Ajzen, 1991) states that people form attitudes or evaluations towards a health behaviour as well as subjective norms. Subjective norms reflect perceptions of whether relevant others value a health behaviour and whether people want to comply with that. Together, attitudes and subjective norms shape the formation of behavioural intentions, which are a proxy for actual behaviour. Combined approaches have also included constructs from both HBM and TPB to predict different health behaviours (Gerend & Shepherd, 2012; Huang et al., 2020). Building on this combined approach, the current study tests whether a humorous health campaign can improve WSW’s binge drinking determinants. Specifically, we investigate effects on the perceived risk of binge drinking (Chen, 2018), attitudes towards binge drinking (Norman & Conner, 2006), and behavioural intentions around binge drinking (Jang et al., 2013).

A classic approach to health campaigns is to instil fear in order to prevent risky and unhealthy behaviours. Such fear appeals are abundant in health campaigns, as illustrated by the use of health warnings and unpleasant images on cigarette packages to remind smokers of the negative impacts of smoking. According to the protection motivation theory (PMT), people who are faced with such a fear appeal engage in two processes - threat appraisal and coping appraisal - to decide whether they should change their behaviour in order to protect themselves against this risk (Rogers, 1975, 1983). Threat appraisal refers to perceptions of the probability and severity, as well as the perceived rewards of continuing one’s current behaviour. Coping appraisal consists of perceptions of self-efficacy (whether a person can engage in the adaptive behaviour), response efficacy (whether the adaptive behaviour sufficiently protects against the threat), and perceived costs of the adaptive behaviour (including non-material costs such as the effort that adapting one’s behaviour requires). If people are faced with a fear appeal, this may trigger high threat and coping appraisals and, hence, motivate people to change their behaviour.

This implies that fear appeals do not always exert the intended effects on health-related behaviours; inducing threat only results in beneficial behavioural change if those targeted by the campaign feel efficacious – that is, if they feel that they can actually do something to reduce the threat (Peters et al., 2013). Being presented with threatening health-promoting information can evoke defensive reactions, motivating people to dismiss or disregard threatening information (Van ‘t Riet & Ruiter, 2013). Moreover, meta-analytic evidence indicates that healthy behaviours may be stimulated more by campaigns increasing efficacy perceptions than by fear appeals (Ruiter et al., 2014). Campaign messages and behaviour change attempts are frequently designed to instil fear while the crucial coping appraisal is overlooked (Roberto, Mongeau, & Liu, 2018). Although merely adopting positive appeals instead of fear appeals may be too simplistic (Cho & Salmon, 2007), a growing body of evidence points to the potential value of humour in health campaigns. Research on humour in alcohol advertisements suggests that humour increases the extent to which people talk about a message as well as the valence of these conversations, which in turn influences how they think about alcohol (Hendriks & Strick, 2020). Applied to anti-alcohol health campaigns, this implies that humour may also increase the informal spreading of a campaign. Furthermore, women in particular are more readily persuaded by humorous health messages that lack fear appeal (Hendriks & Janssen, 2018). Thus, humour without fear appeal may be particularly effective in health campaigns targeting women – as is our current approach.

Although humour was traditionally viewed as appropriate for marketing campaigns on non-durable and low-involvement consumption products, more recently the application of and research on humour in more serious areas of behaviour change such as health campaigns have increased (Weinberger & Gulas, 2019). Indeed, recent eye-tracking evidence indicates that people look longer and more frequently at health messages if these contain humour, suggesting that humour can counteract the tendency of target audiences to look away from confrontational health messages (Brigaud et al., 2021). Humour may produce behavioural change through different mechanisms. Besides attracting attention, humour has also been proposed to release tensions, to enhance source credibility, or to produce a good mood and, hence, reduce counterarguing (for an overview, see Walter et al., 2018). A meta-analysis by Walter et al., (2018) furthermore suggests that both aspects of the humour itself as well as the domain in which it is used influence the effectiveness of humour in changing attitudes and behaviours. That is, humour has weaker effects on persuasion in health-related domains, presumably because health-related decisions are usually longer and better thought through and less ad hoc than, for instance, consumer decisions. Moreover, they concluded that humour effects on persuasion seem to follow an inverted U shape; Humour should be funny but not too funny in order to affect people’s attitudes and decisions. And finally, both people who have high and low involvement tend to be more strongly persuaded by humour that is related to the persuasive message, whereas unrelated humour only seems to work with people who have low involvement. In other words, the humour subject should be aligned with the persuasive message.

## Humour subject: joke about binge drinking or about staying sober

Humour related to a campaign to decrease binge drinking can take the form of a joke about binge drinking or about staying sober. Humour about excessive drinking might backfire if it causes positive conversations about alcohol consumption. Specifically, a recent study using explicit instructions to talk positively or negatively about binge drinking showed that positive conversational valence can induce less healthy binge drinking attitudes and perceived behavioural control (Hendriks et al., 2020). Indeed, public health messages most effectively facilitate adaptive behaviour change if they, among other aspects, (1) clearly define the targeted social group, (2) affirm the group’s identity, and (3) include complementary social norms (Neville et al., 2021). For the current context this suggests that the cartoon should depict a female-female romantic couple who are staying sober rather than drinking excessively during the COVID-19 pandemic.

However, other findings suggest that these effects may reverse depending on the health campaign’s audience. That is, negative-restrictive slogans such as “Don’t drink” increase the perceived risk of excessive drinking and decrease drinking intentions among underage moderate drinkers, but underage binge drinkers show the opposite effects (Lee, M. J. & Chen, 2013). Binge drinkers may feel threatened by the health-promoting message and hence disregard it (Van ‘t Riet & Ruiter, 2013) but humour that portrays the threat as absurd can reduce anxiety (Ford et al., 2017). One may infer that a cartoon on binge drinking may render a health campaign to reduce binge drinking less threatening and, hence, more effective among binge drinkers than a cartoon on staying sober. Indeed, cartoons making fun of problematic behaviour can effectively change behaviour: Female students who saw a cartoon that ridicules non-prepared students outperformed female students who read about non-prepared students being insulted or who were merely reminded of the importance to read study materials (Bryant et al., 1981). Furthermore, humour about others’ heavy drinking can reduce perceived acceptability and binge drinking intentions among binge drinkers who are not highly personally invested in alcohol consumption (Lee, J. Y., Slater, & Tchernev, 2015). To summarize, the effects of the subject of humour (binge drinking versus staying sober) on a health campaign’s effectiveness depend on several factors. We therefore set out to explore whether a humorous health campaign works better – that is, increases sharing of campaign materials and perceived risk of binge drinking, and decreases binge drinking attitudes and intentions—if the punchline of a cartoon is about staying sober, compared to when it is about binge drinking.

## The current research

The aim of the current study is to investigate effects of humour in a health campaign to reduce WSW’s binge drinking during COVID-19. This social context of WSW in a pandemic is particularly relevant because COVID-19 related stressors such as worrying and loneliness may worsen alcohol problems among this group that is already prone to heavy drinking. We zoomed in on two aspects of humour by manipulating two elements in cartoons. Firstly, we exploratively compared cartoons about binge drinking versus about staying sober. Secondly, we created one version of each cartoon depicting a male-female couple—thereby aligning with the heteronormative approach of many health campaigns—and another version depicting a female-female couple—thereby adapting to the WSW target group. Based on the literature discussed above we expected that a humorous health campaign depicting a female-female couple would increase perceived inclusiveness of the campaign (H1) and elicit more positive and less negative affective reactions (H2). We independently assess multiple affective reactions (e.g., amusement, anger), in line with existing notions that individual positive and negative reactions to humour can vary independently (Heintz, 2020; Ruch, 1988, 1992; Ruch & Forabosco, 1996; Warren et al., 2019). Furthermore, we hypothesized that depicting a female-female couple in a humorous campaign would lead to more willingness of WSW to share the campaign materials (H3), and improve their binge drinking determinants – as indicated by higher perceived risk (H4a), more negative attitudes (H4b), and lower behavioural intentions to engage in binge drinking (H4c). Moreover, based on the effects of social identification on group commitment (Ellemers et al., 1997) and motivated behaviours to improve the group’s collective conditions (Van Zomeren et al., 2008), we expected that these beneficial effects of depicting a female-female romantic couple would be larger for people who identify more strongly with WSW and weaker among low WSW identifiers, who are presumably less sensitive to whether the cartoon depicts a male-female or female-female romantic couple (H5).

## Method

### Participants and design

Participants were recruited through Dutch organizations that affiliated with the LGBTQ + community^1^ and through snowballing via Facebook starting in the second author’s personal network (no compensation for participants), as well as among first-year psychology students at the University of Groningen (for partial course credit). One-hundred-and-twenty-seven self-categorized WSW (106 women, one non-binary, 20 missing values regarding gender; 47 lesbian, 42 bisexual, 13 *other* [e.g., pansexual, queer], four heterosexual, 1 rather not say, 20 missing values regarding sexual orientation) consented to participate and were equally distributed over the conditions of a 2 (depicted couple: male-female, female-female) X 2 (humour subject: drinking, staying sober) between-participants design via Qualtrics.^2^ Participants’ age categories ranged from 16–20 years (*n* = 19) to 46–50 years (*n* = 1), most participants were between 21–25 years old (*n* = 45) and 20 were missing values regarding age. Most were single (*n* = 37) or living together (*n* = 35), one was dating exclusively online, 19 dating (also) offline, 15 indicated other (e.g., living apart together), and 20 were missing values regarding relationship status.

The data were collected during the first wave of the COVID-19 pandemic in The Netherlands and the cartoons explicitly referred to lockdown measures. Most participants indicated that they could have a drink together with a close other in their current situation (*n*_yes_=97; *n*_no_=7, 23 missing) but could not visit a drinking establishment (*n*_yes_=35; *n*_no_=68, 24 missing). The prevalence of past-year drinking behaviours ranged from abstinence (*n* = 6) to drinking alcohol every day (*n* = 2; 19 missing), no-one drank four or more drinks every day and 17 never did this in the past year (19 missing).

The study was approved by the Ethics Committee Psychology of the University of Groningen (research code PSY-1920-S-0373). We report all measures and exclusions.

### Materials and procedure

Participants providing their informed consent to participate in a study on attitudes and behaviours concerning alcohol consumption among WSW, and confirmed that they identify as WSW. Next, they read that they would see a message on alcohol consumption; They were instructed to carefully read the text and look at any images, and informed that they would be asked some questions about this information later.

#### Manipulations of depicted couple and humour subject

Participants saw a brief campaign message addressing excessive alcohol consumption: “Too many people are suffering from alcohol abuse. People may have diverse reasons for why they drink too much. For instance, alcohol abuse may result from feeling lonely or feeling that you are not accepted for who you are. Let’s support each other, this is a problem that we can only fight together!”, accompanied by a cartoon. We manipulated the romantic couple that was depicted (female-female, male-female) and the subject of the humour (excessive drinking, staying sober) in the cartoon.

The cartoons were titled “Effects of self-quarantine”. The excessive drinking cartoons consisted of eight panes. The first pane read “Now that the bars are closed, we stay at home”. The remaining seven panes all showed identical pictures of a drinking couple, in each pane accompanied by a different weekday and a different reason justifying that this was a good day for drinking (e.g., “We deserve a few drinks to fight the Monday blues.”; redressing an existing cartoon to the context of drinking couples during the COVID-19 pandemic). One version of this cartoon depicted a male-female couple and the other version depicted a female-female couple. Thus, the COVID-19 situation was linked with excessive drinking in these cartoons.

The staying sober cartoons consisted of two panes. The left pane read “At home, coping with life out there. Before:” and showed a drunk couple sitting on their couch surrounded by emptied wine bottles and glasses. The right pane read “At home, coping with life out there now” and showed the same picture but here the emptied wine bottles and glasses were replaced with toilet paper rolls. Again, one version depicted a male-female couple and the other a female-female couple. This cartoon fitted in a repertoire of jokes at the time on people apparently prepping for the COVID-19 pandemic by hoarding toilet paper and linked the COVID-19 situation to staying sober.

Each participant saw only one of these four cartoons (excessive drinking or staying sober cartoon, depicting a male-female or female-female couple). After indicating whether they took sufficient time for looking at the message (both the cartoon and the text) they continued to the questionnaire.

#### Measures

Unless mentioned otherwise, all items were measured on a -3 (strongly disagree) to 3 (strongly agree) scale. Participants were asked to quickly rate the content of the alcohol consumption message, first the cartoon and then the text, on how amused, outraged, sad, entertained, angry, hopeless, and empowered it made them feel (cf. Thomas et al., 2020). Because discrete affective reactions to humour can vary independently (Heintz, 2020; Ruch, 1988, 1992; Ruch & Forabosco, 1996; Warren et al., 2019) we differentiated four affective reactions (amusement, anger, sadness, empowerment). Amused and entertained assessed participants’ amused reactions to the cartoon, Spearman-Brown *r* = .724, *p* < .001, and the text, Spearman-Brown *r* = .532, *p* < .001. Outraged and angry assessed angry reactions to the cartoon, Spearman-Brown *r* = .546, *p* < .001, and the text, Spearman-Brown *r* = .616, *p* < .001. Sad and hopeless assessed sad reactions to the cartoon, Spearman-Brown *r* = .411, *p* < .001, and text, Spearman-Brown *r* = .421, *p* < .001. Empowerment was included as a single-item response to the cartoon and text, respectively. Subsequently, participants indicated to what extent they would talk about the campaign materials with friends / show it to women they know / share it on their social media timeline. Because deleting the third item improved scale reliability, the first two items were combined to measure offline sharing intentions, Spearman-Brown *r* = .717, *p* < .001, and the latter was retained as a single-item measure of online sharing intentions. An additional open-ended question on campaign sharing through other means was not analysed because of lack of response.

Participants were instructed to answer the following questions imagining their life being “back to normal” as before the COVID-19 pandemic. Perceived risk of binge drinking was computed as the average of perceived probability of negative consequences “If you binge drink, how likely is it that you will get sick, get hurt, or get into trouble?” and perceived severity of negative consequences “If you got sick, got hurt, or got into trouble from binge drinking, how serious do you think it would be?” from − 3 (far below average) to 3 (far above average), Spearman-Brown *r* = .615, *p* < .001 (Chen, 2018). Attitudes towards binge drinking were assessed with five semantic differential scales asking whether going to a bar and consuming 4 or more alcoholic drinks on 1 occasion during the next week is bad-good, foolish-wise, harmful-unharmful, pleasant-unpleasant, enjoyable-unenjoyable (Cronbach’s α = 0.836; Norman & Conner 2006). Three items assessed own binge drinking intentions in the next week / month / year (Jang et al., 2013) and three similar items intentions to let a close other binge drink.^3^ These six items formed one scale of behavioural intentions around binge drinking (Cronbach’s α = 0.914).

Subsequently, two items measured the prevalence of alcohol use and binge drinking on a 10-point scale from *every day* (left-hand anchor) to *1–2 times* (right-hand anchor) with an additional scale point indicating *never* (McCabe et al., 2006). Social identification with WSW was measured with a single-item measure (Postmes et al., 2013). Perceived inclusion was measured as “In this campaign message against alcohol abuse (cartoon + text at the start of this study), the WSW community is: included / addressed / given a voice” (Cronbach’s α = 0.879). Participants provided their gender, sexual orientation, relationship status, age (in 5-year categories), and indicated in two items whether or not their current circumstances allowed to have a drink with a close other and to visit a bar. In the debriefing phase, participants were also provided the possibilities to leave comments about the study and to copy a link for further distributing the questionnaire.

## Results

### Preliminary analyses

Two participants indicated that they had not taken sufficient time to look at the stimulus materials. Visual inspection revealed no deviating response patterns and Mahalanobis distance indicated no multivariate outliers with *p* < .001. Hence these participants’ data were included in the analyses reported here.

Seventeen participants indicated that they never engaged in binge drinking during the past 12 months; Four who saw the excessive drinking cartoon depicting a female-female couple, three who saw this same cartoon depicting a male-female couple, and five participants in each of the two remaining conditions with the staying sober cartoon. There were no significant differences between conditions in the binge drinking frequencies of participants who did binge drink during the past 12 months. That is, an ANOVA with depicted couple (female-female, male-female) and humour subject (excessive drinking, staying sober) as predictors and binge drinking frequency as outcome revealed no significant main effects of depicted couple, *F*(1,87) = 2.497, *p* = .118, η_p_^2^ = 0.028, humour subject, *F*(1,87) = 0.418, *p* = .520, η_p_^2^ = 0.005, nor a couple X humour interaction, *F*(1,87) = 0.778, *p* = .380, η_p_^2^ = 0.009. On average, participants scored 6.857 (*SD* = 2.283) on a scale from 1 *every day* to 10 *1–2 times*. Most participants who engaged in binge drinking did so one or two times during the past year (*n* = 17).

Our hypotheses specified WSW identification as a moderator. In all conditions except the male-female excessive drinking condition, WSW identification was negatively skewed (skew values > -2.465, *SE*_skew_ > 0.447, *p* < .001) and leptokurtic (kurtosis values > 7.149, *SE*_kurtosis_ > 0.872, *p* < .001). Therefore, we used bootstrapping with 1,000 samples. An ANOVA with depicted couple (female-female, male-female) and humour subject (excessive drinking, staying sober) as predictors and WSW identification as outcome revealed no significant main effects of depicted couple, *F*(1,103) = 0.129, *p* = .720, η_p_^2^ = 0.001, humour subject, *F*(1,103) = 0.018, *p* = .895, η_p_^2^ < 0.001, nor a couple X humour interaction, *F*(1,103) = 0.001, *p* = .975, η_p_^2^ < 0.001. Furthermore, WSW identification was significantly above the scale midpoint, *t*(106) = 21.160, *p* < .001 (bootstrapped *M*_difference_ = 2.355, 95% CI[2.122, 2.561]). Thus, participants identified rather strongly with WSW across conditions.

### Main analyses

Descriptive statistics for all outcome variables in the four manipulated experimental conditions of the 2 (humour subject: excessive drinking, staying sober) X 2 (depicted couple: male-female, female-female) design are displayed in Table 1.

**Table 1:** Estimated marginal means [ 95% CIs] for all outcome variables, by humour subject (excessive drinking, staying sober) and depicted couple (male-female, female-female). Means are estimated at the mean of WSW identification

Outcome	Excessive drinking			Staying sober	
	**Male-female**	**Female-female**		**Male-female**	**Female-female**
Perceived inclusion	-0.143 [-0.756, 0.470]	0.394 [-0.208, 0.996]		-0.950 [-1.564, -0.337]	0.977 [0.364, 1.590]
Affective reactions to cartoon					
Amusement	0.402 [-0.129, 0.932]	0.443 [-0.078, 0.964]		-0.650 [-1.181, -0.119]	0.815 [0.284, 1.345]
Anger	-0.709 [-1.249, -0.170]	-1.035 [-1.565, -0.506]		-0.737 [-1.277, -0.197]	-1.485 [-2.024, -0.945]
Sadness	-0.082 [-0.642, 0.477]	-0.459 [-1.009, 0.090]		-0.160 [-0.720, 0.400]	-0.326 [-0.885, 0.233]
Empowerment	-1.316 [-1.971, -0.660]	-1.059 [-1.703, -0.416]		-1.067 [-1.723, -0.410]	-0.718 [-1.373, -0.062]
Affective reactions to text					
Amusement	-1.048 [-1.581, -0.515]	-0.556 [-1.080, -0.033]		-0.654 [-1.187, -0.120]	0.335 [-0.198, 0.868]
Anger	-0.611 [-1.173, -0.049]	-0.994 [-1.546, -0.443]		-0.436 [-0.998, 0.126]	-1.117 [-1.679, -0.556]
Sadness	0.034 [-0.519, 0.588]	-0.329 [-0.872, 0.215]		0.216 [-0.338, 0.770]	-0.185 [-0.739, 0.368]
Empowerment	-0.603 [-1.289, 0.083]	-1.038 [-1.711, -0.364]		-0.557 [-1.244, 0.130]	-0.227 [-0.914, 0.459]
Sharing intentions					
Offline	-0.210 [-0.895, 0.475]	-0.294 [-0.966, 0.379]		− 0.0698 [-1.383, -0.012]	-0.502 [-1.187, 0.183]
Online	-1.720 [-2.363, -1.077]	-1.968 [-2.600, -1.337]		-1.890 [-2.533, -1.246]	-1.427 [-2.070, -0.784]
Binge drinking determinants					
Perceived risk	-0.457 [-1.119, 0.205]	-0.682 [-1.332, -0.032]		-0.057 [-0.720, 0.606]	-0.459 [-1.121, 0.203]
Attitudes	-0.177 [-0.693, 0.338]	-0.510 [-1.016, -0.003]		-0.076 [-0.592, 0.440]	-0.531 [-1.046, -0.015]
Behavioural intentions	0.898 [0.226, 1.571]	1.376 [0.716, 2.036]		0.885 [0.212, 1.558]	0.999 [0.327, 1.671]

#### Perceived inclusion

**Fig. 1:**
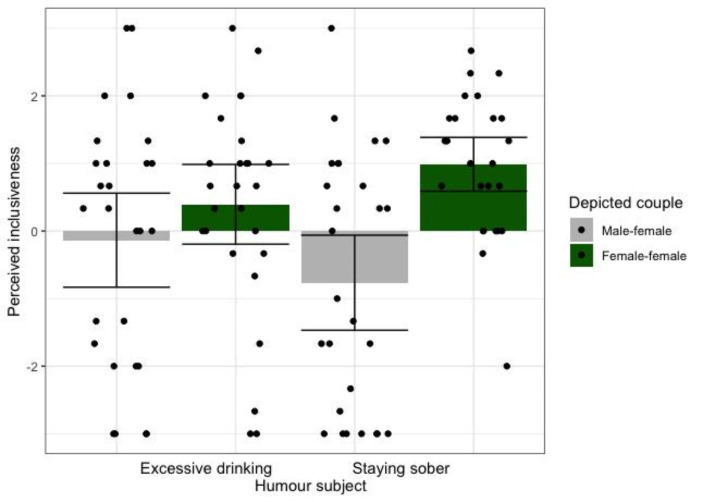
Condition means (95% CI error bars) and raw data points, showing that WSW feel more included in the humorous health campaign when it depicts a female-female couple than when it depicts a male-female couple – particularly when the humour concerns staying sober (compared to excessive drinking)

To test the hypotheses that WSW feel more included in a health campaign depicting a female-female couple than in a male-female campaign, and that this effect is stronger for people who more strongly identify with WSW, we conducted an ANCOVA with depicted couple (female-female, male-female), humour subject (excessive drinking, staying sober), and mean-centered WSW identification as predictors and perceived inclusion as outcome. Because not all variables were normally distributed, we used bootstrapping with 1,000 samples. As expected, participants in the female-female condition felt more included (estimated marginal mean [*EMM*] = 0.687, 95% CI [0.306, 1.002]) than in the male-female condition (*EMM* = -0.465, 95% CI [-0.944, -0.062]), *F*(1,99) = 13.599, *p* < .001, η_p_^2^ = 0.121. A significant depicted couple X humour subject interaction indicated that the main effect of depicted couple on perceived inclusion was driven by the staying sober cartoons, *F*(1,99) = 3.956, *p* = .049, η_p_^2^ = 0.038 (see Fig. 1). WSW identification did not moderate the effect of depicted couple, *F*(1,99) = 1.849, *p* = .177, η_p_^2^ = 0.018, nor were there any other significant main effects or interactions on perceived inclusion, all *F*s < 1.313, *p*s > 0.254.

#### Affective reactions

We furthermore hypothesized that WSW react more positively to a health campaign that depicts a female-female couple than one that depicts a male-female couple, and that this effect is stronger for people who more strongly identify with WSW. These hypotheses were tested in two MANCOVAs with depicted couple (female-female, male-female), humour subject (excessive drinking, staying sober), and mean-centered WSW identification as predictors, and as outcomes affective reactions (amusement, anger, sadness, empowerment) to the cartoon and, respectively, the text of the health campaign. In the first analysis, deviations from normality were again accounted for by bootstrapping.

**Fig. 2:**
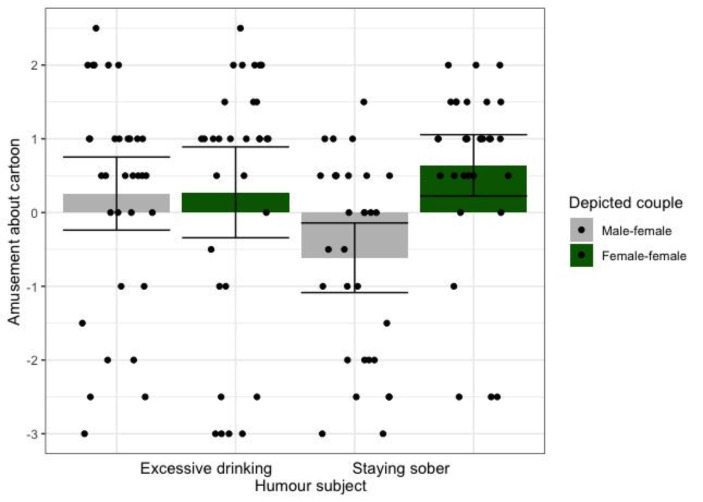
Condition means (95% CI error bars) and raw data points, showing that WSW are more amused by the cartoon when it depicts a female-female couple than when it depicts a male-female couple, particularly when the humour concerns staying sober (compared to excessive drinking)

**Fig. 3:**
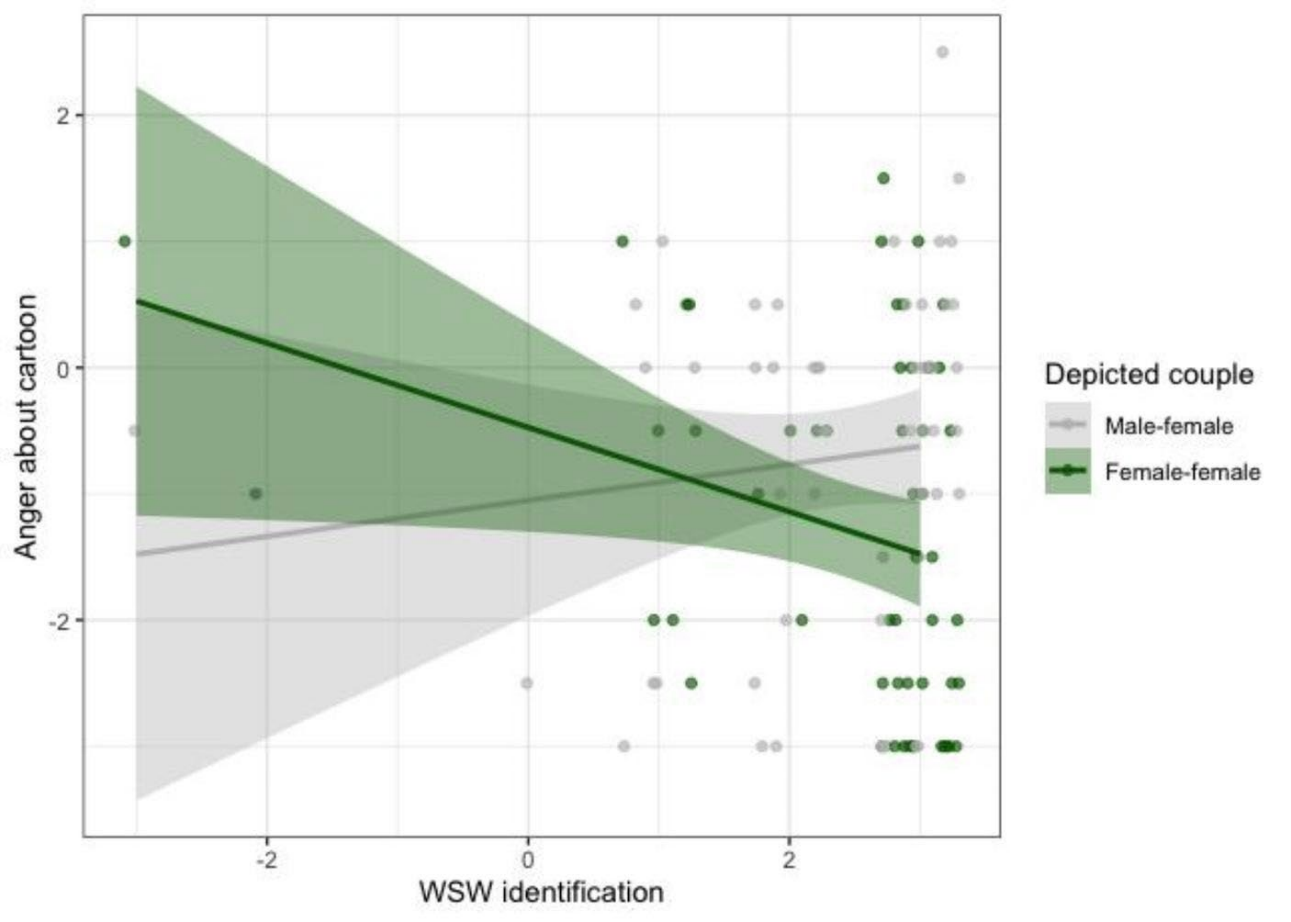
Raw data points (with 0.2 horizontal jitter) and fitted lines, showing that WSW identification increases anger about the male-female cartoon and decreases anger about the female-female cartoon. Note: Most data points are on the right-hand side of the plot, hence the finding that the female-female cartoon elicited less anger than the male-female cartoon

As hypothesized, the first MANCOVA revealed that participants who saw the cartoon depicting a female-female couple felt more amused (*EMM* = 0.628, *SE* = 0.187, 95% CI [0.256, 0.999]) than participants who saw the male-female couple (*EMM* = -0.086, *SE* = 0.186, 95% CI [-0.454, 0.282]), *F*(1,99) = 7.333, *p* = .008, η_p_^2^ = 0.069. Again, a significant depicted couple X humour subject interaction indicated that the main effect of depicted couple on amusement was driven by the staying sober cartoons, *F*(1,99) = 7.888, *p* = .006, η_p_^2^ = 0.074 (see Fig. 2). Moreover, participants who saw the cartoon depicting a female-female couple felt less anger (*EMM* = -1.264, *SE* = 0.189, 95% CI [-1.639, -0.889]) than participants who saw the male-female couple (*EMM* = -0.718, *SE* = 0.187, 95% CI [-1.090, -0.347]), *F*(1,99) = 4.215, *p* = .043, η_p_^2^ = 0.041. A significant depicted couple X WSW identification interaction on anger indicated that this effect increased with WSW identification, *F*(1,99) = 6.231, *p* = .014, η_p_^2^ = 0.059 (see Fig. 3). There were no other significant main effects or interactions on affective reactions to the cartoon, *F*s < 3.779, *p*s > 0.054.

**Fig. 4:**
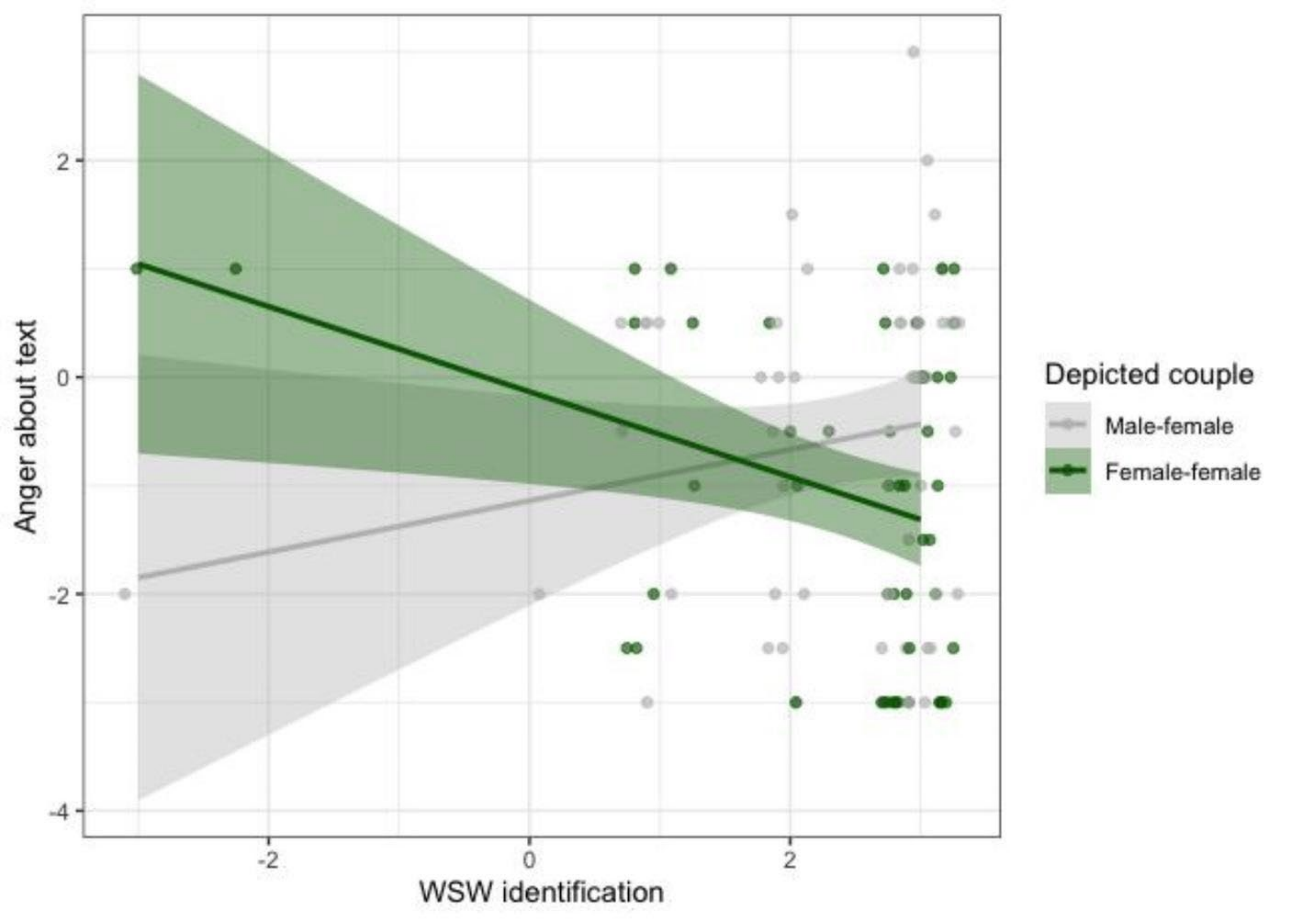
Raw data points (with 0.2 horizontal jitter) and fitted lines, showing that WSW identification increases anger about the health campaign text when it portrays a male-female cartoon and decreases anger about the health campaign text when it portrays a female-female cartoon

The second MANCOVA showed that participants in the female-female couple condition were more amused by the health campaign’s text (*EMM* = -0.112, *SE* = 0.187, 95% CI [-0.484, 0.260]) than in the male-female condition (*EMM* = -0.862, *SE* = 0.187, 95% CI [-1.234, -0.490]), *F*(1,98) = 8.053, *p* = .006, η_p_^2^ = 0.076. Moreover, participants who saw the staying sober cartoon were more amused about the health campaign’s text (*EMM* = -0.161, *SE* = 0.189, 95% CI [-0.536, 0.215]) than participants who saw the excessive drinking cartoon (*EMM* = -0.813, *SE* = 0.185, 95% CI [-1.181, -0.445]), *F*(1,98) = 6.110, *p* = .015, η_p_^2^ = 0.059. WSW identification negatively predicted amusement about the text, *F*(1,98) = 10.324, *p* = .002, η_p_^2^ = 0.095. Finally, a significant depicted couple X WSW identification interaction on anger at the health campaign’s text showed that the more strongly participants identified with WSW the more angry were about the male-female (relative to female-female) health campaign’s text, *F*(1,98) = 8.886, *p* = .004, η_p_^2^ = 0.083 (see Fig. 4). This latter interaction effect and the main effect of depicted couple mimicked the affective reactions to the cartoon. There were no other significant effects on affective reactions to the text, *F*s < 3.546, *p*s > 0.062.

#### Campaign-sharing intentions and binge drinking determinants

In a MANCOVA with depicted couple (female-female, male-female), humour subject (excessive drinking, staying sober), and mean-centered WSW identification as predictors and offline and online sharing intentions as outcomes, deviations from normality were again accounted for by bootstrapping. Results indicated that WSW identification negatively predicted offline sharing intentions, *F*(1,99) = 5.111, *p* = .026, η_p_^2^ = 0.049. There were no other significant main effects or interactions, *F*s < 3.347, *p*s > 0.069. Thus, the manipulations did not affect participants’ intentions to share campaign materials, but there was a main effect of WSW identification.

A MANCOVA with depicted couple (female-female, male-female), humour subject (excessive drinking, staying sober), and mean-centered WSW identification as predictors and perceived risk, attitudes, and binge drinking intentions as outcomes revealed that the more strongly people identified with WSW, the more positive their attitudes towards binge drinking, *F*(1,98) = 5.031, *p* = .027, η_p_^2^ = 0.049, but the lower their actual binge drinking intentions, *F*(1,98) = 5.001, *p* = .028, η_p_^2^ = 0.049. There were no further significant main effects or interactions, *F*s < 3.454, *p*s > 0.065. Thus, the manipulations did not affect participants’ binge drinking determinants, but there were some main effects of WSW identification.

## Discussion

With the current research on a humorous health campaign against binge drinking, we aimed to investigate the effects of depicting a female-female romantic couple in a cartoon (versus a male-female couple) and of the humour subject (cartoon joking about excessive drinking versus about staying sober) on WSW’s perceived inclusion, affective reactions to the campaign, campaign-sharing intentions, and binge drinking determinants. Overall, we expected that a health campaign depicting a female-female couple would be received more positively and would be more effective among this target group. That is, we hypothesized that depicting a female-female couple in a humorous campaign would increase perceived inclusiveness of the campaign (H1), elicit more positive and less negative affective reactions (H2), lead to more willingness of WSW to share the campaign materials (H3), and improve their binge drinking determinants – as indicated by higher perceived risk (H4a), more negative attitudes (H4b), and lower behavioural intentions to engage in binge drinking (H4c). Finally, we hypothesized that these effects would be larger for high WSW identifiers compared to low WSW identifiers (H5). Results indicated support on various – although not all – measures. We will first briefly recap the main findings, before turning to a more in-depth discussion.

### Main findings of the current study

WSW perceived that their group was more included in the humorous health campaign when it showed a cartoon depicting a female-female couple rather than a male-female couple. This positive effect of depicting a female-female couple on perceived inclusiveness particularly emerged in the cartoons about staying sober. Similarly, WSW were more amused about the female-female cartoon and text than about the male-female cartoon. The cartoon amusement was again particularly affected by the depicted couple when the humour concerned staying sober rather than binge drinking. Moreover, the female-female cartoon elicited less angry responses than the male-female cartoon – especially among high identifiers. The text of the health campaign elicited less anger among high identifiers when the cartoon depicted a female-female rather than male-female couple. Furthermore, high identifiers were less amused about the health campaign text and less likely to share the campaign materials offline. They had more positive attitudes towards binge drinking but lower binge drinking intentions than low identifiers. Finally, participants who saw the staying sober cartoon indicated to be more amused about the health campaign’s text than participants who saw the excessive drinking cartoon.

To summarize, although the (very brief) health message did not affect binge drinking determinants, the humorous health campaign depicting a female-female couple was received more positively than when the cartoons depicted a male-female couple. That is, the hypothesized beneficial effects of depicting a female-female rather than male-female couple were supported by significant results indicating higher perceived inclusiveness, more amused and less angry responses to the humorous cartoon and the campaign text. Evidence for the hypothesis that these effects would be stronger among participants with high WSW identification was weak; WSW identification only moderated the effects of depicted couple on angry responses to the cartoon and text. The cartoon’s humour subject (excessive drinking versus staying sober) moderated the effects of depicted couple on perceived inclusiveness and amusement about the cartoon. The hypothesized beneficial effects of depicting a female-female couple were not found on empowerment, sadness, sharing intentions, and binge drinking determinants (perceived risk, attitudes, and behavioural intentions). Nor did any of these outcomes show a *negative* effect of depicting a female-female couple in the humorous cartoon.

### Depicting female-female versus male-female couples in health campaigns

The results suggest that adding a humorous cartoon depicting a female-female romantic couple to an anti-alcohol health campaign message induces positive affective reactions (more amusement and less anger) to the humour in particular as well as the health campaign message more generally. The current research compared humorous cartoons depicting a female-female couple to a very close comparison condition, in which exactly the same stimulus was presented with the only difference that the illustration depicted a male-female couple. This very conservative test nonetheless provided proof of concept that a humorous cartoon depicting a female-female couple not only increases a health campaign’s perceived inclusiveness but also enhances affective reactions in the WSW target group. This is in line with existing findings that diversity messages that explicitly mention the cultural majority increase perceived inclusion among job applicants from a cultural majority background (Jansen et al., 2015). Although feeling included may be especially important for minorities such as LGBTQ + people (Bagci et al., 2020), people in general want to feel positively about the groups they identify with (Postmes & Branscombe, 2010). This may be particularly true of strong identifiers, who tend to be more committed to their group (Ellemers et al., 1997) and aim to improve their group’s collective conditions (Van Zomeren et al., 2008). This may explain why WSW react positively to and feel included in a health campaign that depicts a female-female couple.

These findings extend existing evidence by illustrating that, although humour about a marginalized group can have negative consequences (Ford et al., 2020; Hodson & MacInnis, 2016), depicting such a target group in humour can instead elicit positive affect and perceptions of inclusiveness. An obvious explanation for why the current cartoons did not show negative effects on WSW may be that the humour revolved around another subject (i.e., excessive drinking, staying sober) than the core characteristic of sexual orientation that distinguishes WSW from other social categories. Nonetheless, these findings debunk the stereotype that WSW lack sense of humour (Geiger et al., 2006) and instead point to the potential of humour depicting a female-female romantic couple to reach this target group. This is an important contribution in a time in which pervasive stressors such as the COVID-19 pandemic push individuals – and particularly WSW, who are already prone to excessive alcohol use (Hyde et al., 2009; McCabe et al., 2010) – towards alcohol as self-medication coping (Cerezo et al., 2021). An anti-alcohol health campaign using humorous cartoons that depict female-female couples may thus constitute a promising means to meet the demand for a specifically targeting high-risk groups (Shields et al., 2016) and in particular vulnerable LGBTQ + groups (Drabble & Eliason, 2021).

The current research differentiated between a variety of affective responses to the humorous content. The significant effects on amusement and anger are consistent with existing literature highlighting these two as predominant affective reactions to humorous content (e.g., Thomas et al., 2020). Moreover, the lack of concomitant effects on affective reactions of sadness and empowerment resonates with well-established conclusions that separate positive and negative reactions to humour can vary independently (Heintz, 2020; Ruch, 1988, 1992; Ruch & Forabosco, 1996; Warren et al., 2019).

The current manipulations did not significantly affect any of the binge drinking determinants (perceived risk, attitudes, and behavioural intentions). This lack of effect seems to contrast existing demonstrations of the persuasive role of ridicule in motivating behavioural change (Bryant et al., 1981; Lee et al., 2015). This may be explained by the health message itself, which was quite short and not very concrete. An alternative explanation may be that earlier studies involved making fun of more lifelike other people or addressed the audience more directly in the cartoon, whereas the current research featured cartoon protagonists that were not introduced explicitly as an illustration of (some of) the study participants. Alternatively, the content of the cartoons may have prevented the hypothesized reduction in binge drinking intentions. That is, all cartoons included an illustration of a couple engaged in binge drinking. This could have functioned as a descriptive norm indicating that binge drinking is normal – thereby countering the prescriptive norm in the campaign message itself, to help each other in reducing alcohol abuse (Cialdini et al., 1991). Positive psychologists sometimes overlook that humour does not necessarily lead to healthier or more resilient responses yet that it is a multi-faceted construct that can be both adaptive and maladaptive (Kuiper, 2012). This may also explain why there were no consistent significant effects on binge drinking determinants; The effects of humour depend on people’s idiosyncratic humour styles. That is, people with a self-enhancement humour style may use the cartoons to cope with the stress of COVID-19 and/or drinking problems by reframing their situation in a more humorous, light-hearted way, whereas others with a self-defeating humour style may use the cartoons in a self-disparaging way and put themselves down to avoid confrontation with their problems and negative feelings (Ford et al., 2017; Martin et al., 2003).

Existing research on humour and health behaviour change may provide further explanations for the lack of effects on binge drinking determinants. The current research involves deliberate, potentially high-involvement choices (i.e., to change one’s lifestyle concerning alcohol consumption or to help another person achieve this). These kinds of health behaviour changes typically evoke resistance (Blondé & Falomir-Pichastor, 2021). Similarly in line with the current results, a recent meta-analysis showed only weak effects of humour on persuasion, attitudes, and behavioural intentions, especially in the health domain (Walter et al., 2018). According to the elaboration likelihood model, people can process information via the central route, based on critical deliberation and careful consideration of the content, or via the peripheral route, based on quick and dirty heuristics and rules of thumb (Petty & Cacioppo, 1986). When a health message is paired with humorous content, as in the current research, recipients are required to process and understand both the humour and the health message itself. Pairing with humour can make people less able or less motivated to carefully process the message itself, and it can even signal to people that careful processing is not necessary in the current context (Young, 2008). This implies that humour can lead to peripheral route processing or even straightforward discounting of the health message content.

The lack of a negative effect indicates that a funny cartoon (even when explicitly depicting binge drinking behaviour) does at least not seem to worsen binge drinking determinants. Solitary viewing of humorous cartoons about binge drinking thus does not appear to have the same deleterious effects as fun conversations about binge drinking (Hendriks et al., 2020). Nonetheless, people’s reactions to humour may be swayed by the responses of others around them (Thomas et al., 2020) so future research should further investigate potential effects of co-present others’ reactions. In the current context, it would be particularly interesting to explore effects of WSW versus non-WSW others’ reactions.

The finding that people were less amused by the cartoon about excessive drinking (i.e., making fun of a real-life problem) than by the cartoon about staying sober may reflect a mere difference in funniness of the cartoons. However, this pattern could also be explained by people’s reluctance to be amused by current real-life problems compared to problems that belong to the (recent) past (McGraw et al., 2014). The findings are consistent with the notion that psychological distance facilitates displays of amusement about something threatening, whereas psychological closeness facilitates humor when the threat is perceived as relatively minor or benign (McGraw et al., 2012). That is, WSW were more amused by the female-female cartoon (i.e., psychological closeness) than by the male-female cartoon (i.e., psychological distance), but only when the humour subject was benign (staying sober) rather than confronting and threatening (excessive drinking).

### Limitations and future directions

The current study focused on WSW’s reactions to the campaign materials. WSW felt more included and more positive affect when the cartoons depicted a female-female couple compared to a male-female couple. However, a limitation is that in real-life it would be very difficult to spread campaign materials exclusively among a certain sub-group of the general population. Future research should therefore compare the effects with different audiences that do not identify as WSW. Making fun of another group’s suffering can be socially accepted under certain situations (Leach et al., 2003). Given the higher vulnerability and prevalence of heavy drinking among WSW (Hyde et al., 2009; McCabe et al., 2010), alcohol problems may be a negative stereotype about WSW. Although mere exposure to disparagement humour may not sway people’s attitudes and stereotypes concerning the target group (Olson et al., 1999), actively telling disparagement humour can invigorate negative stereotypes (but not attitudes; Maio et al., 1997). Indeed, especially people who already feel negatively about a target group may feel safe to express their negativity in a context in which that target group is the subject of disparaging jokes (Ford et al., 2001). This raises the question whether cartoons depicting a female-female couple excessively drinking together may have negative consequences for others’ reactions to WSW. Because of the high prevalence of anti-LGBTQ + prejudice and discrimination, this is an important venue for follow-up research.

Another potential limitation is that the humorous campaign materials were shown to participants in isolation. However, co-present others’ reactions with amusement or anger can influence whether the cartoon depicting a female-female couple excessively drinking is seen as just a joke or as a moral violation that should be confronted (cf. Thomas et al., 2020). Given that WSW are disproportionately suffering from excessive drinking, such humour about a serious issue may be perceived as inappropriate. The (social) appraisal and interpretation of humour thus seems important to explore further. Evidence on ethnic humour (Saucier et al., 2016) underscores the relevance of this issue, because prosocial humour meant to diminish problematic behaviour can unintentionally fuel it if the humour is interpreted as condoning the problematic behaviour. Further research could study more systematically whether excessive drinking humour can reinforce binge drinking culture whereas subversive humour can subvert this problem – mirroring effects of rape humour on normalization of rape and rape culture (Strain et al., 2016).

Future research could further investigate humorous health campaigns as a double-edged sword, potentially both improving health by increasing the persuasiveness of health messages and simultaneously helping people to better cope with adversity. Adding explicit instructions on what to do with the humorous cartoons could provide more insight into the mechanisms at work in determining the effectiveness of such health messages. Ford and colleagues (2017) investigated self-enhancing humour, defined as finding amusement in the absurdity of a negative situation (in their research a surprise math test), and self-defeating humour, defined as making fun of one’s own shortcomings to lessen the sting of failing. They found that self-enhancing humour reduces negative emotions compared to no humour and self-defeating humour, only when self-enhancing humour is accompanied by instructions to maintain a positive view during hardship. The current research did not include explicit instructions regarding what to do with the presented humour. However, the current manipulation of humour subject did contain elements of self-enhancing humour (finding amusement in our whole situation’s absurdity by joking about hoarding toilet paper) and, respectively, self-defeating humour (making fun of us finding excuses to drink excessively each day of the week). The findings by Ford et al. suggest that simply adding an explicit instruction can turn self-enhancing humour into a conscious coping strategy and thereby improve resilience.

More generally, there are many theories on humour and amusement (for an overview, see Warren et al., 2021). Open questions that constitute interesting venues for future research entail, for example: Which antecedents of laughter and amusement in particular are good candidates for a humorous health campaign? Which antecedents are useful in which stage of a health campaign? Are different antecedents effective in campaigns promoting healthy behaviour than in campaigns focusing on prevention of illness or risky behaviour? Which antecedents of laughter and amusement are most applicable in health campaigns targeting disadvantaged minority groups in particular?

## Conclusions

A humorous health campaign depicting a female-female couple was received more positively (higher perceived inclusiveness, more amusement, less anger) than when the humorous health campaign depicted a male-female couple. These effects emerged even though the conditions that we compared were identical except for the gender of the cartoon protagonists. The fact that such a conservative test still revealed positive effects of WSW-inclusive compared to hetero-normative humour provides a promising perspective on the value of tailored cartoons in targeting high-risk groups. The current health message did not affect binge drinking determinants, likely because it was too brief and not very concrete. Nonetheless, overall the current findings provide a proof of concept that inclusive humour can benefit the reception of health campaign messages among minorities.

## Footnotes

1 An LGBTQ + student association and a bookstore specialising in feminism, post-colonialism, and LGBTQ + themes both distributed an invitation to our study.

2 Of these, 20 participants did not reach the questionnaire end. Missing data were handled with pairwise deletion. Data of 28 additional participants were excluded because they did not reach the end of the first measures on affective reactions and, hence, could not be meaningfully analysed. To approach an equal distribution of participants over conditions, we used the Qualtrics ‘evenly present elements’ function and manually adjusted the counts whenever we noticed that a participant met the exclusion criterion.

3 The three items concerning participants’ own binge drinking intentions accidentally included 1 additional scale point to the right side of 3 *Extremely likely* in the non-student sample. Because the entire dataset only included six occurrences (*n*_item1_ = 1, *n*_item2_ = 2, *n*_item3_ = 3) these single data points were all set at 3 to improve interpretability of the results.
